# Application of veterinary naturopathy and complementary medicine in small animal medicine—A survey among German veterinary practitioners

**DOI:** 10.1371/journal.pone.0264022

**Published:** 2022-02-28

**Authors:** Ines Stanossek, Axel Wehrend

**Affiliations:** 1 Department of Internal Medicine, Veterinary Clinic for Small Animal Science Frank, Freiburg, Germany; 2 Clinic for Obstretics, Gynaecology and Andrology for Large and Small Animal Science with Veterinary Ambulance, Justus-Liebig-University Gießen, Gießen, Germany; University of Lincoln, UNITED KINGDOM

## Abstract

**Background:**

The international use of and interest in veterinary naturopathy and complementary medicine are increasing. There are diverse modes of treatment, and owners seem to be well informed. However, there is a lack of data that describes the state of naturopathic or complementary veterinary medicine in Germany. This study aims to address the issue by mapping the currently used treatment modalities, indications, existing qualifications, and information pathways. In order to map the ongoing controversy, this study records the advantages and disadvantages of these medicines as experienced by veterinarians. Demographic influences are investigated to describe distributional impacts on using veterinary naturopathy and complementary medicine.

**Methods:**

A standardised questionnaire was used for the cross-sectional survey. It was distributed throughout Germany in a written and digital format from September 2016 to January 2018. Because of the open nature of data collection, the return rate of questionnaires could not be calculated. To establish a feasible timeframe, active data collection stopped when the previously calculated limit of 1061 questionnaires was reached. With the included incoming questionnaires of that day a total of 1087 questionnaires were collected. Completely blank questionnaires and those where participants did not meet the inclusion criteria (were not included, leaving 870 out of 1087 questionnaires to be evaluated. A literature review and the first test run of the questionnaire identified the following treatment modalities: homoeopathy, phytotherapy, traditional Chinese medicine (TCM), biophysical treatments, manual treatments, Bach Flower Remedies, neural therapy, homotoxicology, organotherapy, and hirudotherapy which were included in the questionnaire. Categorical items were processed using descriptive statistics in absolute and relative numbers based on the population of completed answers provided for each item. Multiple choices were possible. Metric data were not normally distributed (Shapiro Wilk Test); hence the median, minimum, and maximum were used for description. The impact of demographic data on the implementation of veterinary naturopathy and complementary techniques was calculated using the Mann-Whitney-U-Test for metric data and the exact Fisher-Test for categorical data.

**Results:**

Overall 85.4% (n = 679 of total 795 non-blank data sets) of all the questionnaire participants used naturopathy and complementary medicine. The treatments most commonly used were complex homoeopathy (70.4%, n = 478), phytotherapy (60.2%, n = 409), classic homoeopathy (44.3%, n = 301) and biophysical treatments (40.1%, n = 272). The most common indications were orthopedic (n = 1798), geriatric (n = 1428) and metabolic diseases (n = 1124). Over the last five years, owner demand for naturopathy and complementary treatments was rated as growing by 57.9% of respondents (n = 457 of total 789). Veterinarians most commonly used scientific journals and publications as sources for information about naturopathic and complementary contents (60.8%, n = 479 of total 788). These were followed by advanced training acknowledged by the ATF (Academy for Veterinary Continuing Education, an organisation that certifies independent veterinary continuing education in Germany) (48.6%, n = 383). The current information about naturopathy and complementary medicine was rated as adequate or nearly adequate by a plurality (39.5%, n = 308) of the respondents of this question. Further, 27.7% (n = 216) of participants chose the option that they were not confident to answer this question and 91 answers were left blank. The most commonly named advantages in using veterinary naturopathy and complementary medicine were the expansion of treatment modalities (73.5%, n = 566 of total 770), customer satisfaction (70.8%, n = 545) and lower side effects (63.2%, n = 487). The ambiguity of studies, as well as the unclear evidence of mode of action and effectiveness (62.1%, n = 483) and high expectations of owners (50.5%, n = 393) were the disadvantages mentioned most frequently. Classic homoeopathy, in particular, has been named in this context (78.4%, n = 333 of total 425). Age, gender, and type of employment showed a statistically significant impact on the use of naturopathy and complementary medicine by veterinarians (p < 0.001). The university of final graduation showed a weaker but still statistically significant impact (p = 0.027). Users of veterinary naturopathy and complementary medicine tended to be older, female, self-employed and a higher percentage of them completed their studies at the University of Berlin. The working environment (rural or urban space) showed no statistical impact on the veterinary naturopathy or complementary medicine profession.

**Conclusion:**

This is the first study to provide German data on the actual use of naturopathy and complementary medicine in small animal science. Despite a potential bias due to voluntary participation, it shows a large number of applications for various indications. Homoeopathy was mentioned most frequently as the treatment option with the most potential disadvantages. However, it is also the most frequently used treatment option in this study. The presented study, despite its restrictions, supports the need for a discussion about evidence, official regulations, and the need for acknowledged qualifications because of the widespread application of veterinary naturopathy and complementary medicine. More data regarding the effectiveness and the mode of action is needed to enable veterinarians to provide evidence-based advice to pet owners.

## Introduction

Naturopathy and complementary medicine are growing fields of interest for veterinarians and pet owners by many international authors [1–5]. Despite this, data on the actual use of these treatment options in veterinary medicine are rare, not specific for small animal science, or have been investigated only for particular treatment modalities [6, 7].

For German-speaking countries (Switzerland, Austria, Germany), Hahn et al. [8] investigated the usage of phytotherapy in small animal medicine. They showed that 79% of 189 participants used this treatment option. In the study of Truls [6], over 60% of the 64 participants in Austria used herbal medicine as a treatment option. Further, Ertl [7] showed that 76% of the 36 participants in Kärnten used phytotherapy for acute diseases. In an American retrospective study from 2005, Shmalberg and Memon [9] showed that 39% of 5195 pets were treated with integrative modalities with the limitation that neither homoeopathy nor chiropractic treatments was offered at the study hospital (Small Animal Clinical Science, University of Florida). For pet owners, Lana et al. [10] showed that homoeopathy, phytotherapy, acupuncture, chiropractic, and Bach flower remedies are used to treat oncologic diseases in their pets.

Data for German human dentistry are presented by Baatsch [11]. In their study, 57% of 250 dentists recommended homoeopathy, 50% osteopathy and, for example, 64% suggested the use of *Arnica montana* for their patients [11]. In a study by Alscher [12] in general and internal medicine as well as orthopaedics, only 15% of 935 participants never used phytotherapy, and 41.7% never used homoeopathy for their patients. Furthermore, Thanner et al. [13] showed that 63% of all participating registered human practitioners used complementary and alternative treatments. Walach and Pietkäinen declare that Germany is a leader when it comes to using these techniques in human medicine [14]. Due to the high adoption of phytotherapy [8] and the public receptiveness to alternate treatment modalities, it is plausible to assume similar leadership by Germany in veterinary medicine.

Nevertheless, considering the different studies on the use of naturopathy and complementary medicine, no comparable data for Germany can be found for small animal science. Therefore, this study aims to deliver insight into the use of naturopathy and complementary medicine in small animal science as a base for the ongoing discussion regarding qualifications, demand and professionals in this field. The objective of this study is not to provide evidence for the validity or effectiveness of treatment options.

In order to foster understanding and for the comparability of study data, a consistent definition of terms is crucial [15, 16]. The application of naturopathy and complementary medicine is part of a worldwide discussion about a grounded definition of terms and contents [15–17]. The term ‘complementary medicine’ was chosen in the context of the “traditional medicine strategy” of the World Health Organization (WHO) [18]. Veterinary complementary treatment modalities are homoeopathy (complex homoeopathy: the use of commercial drugs containing mixtures of homeopathic remedies treating a presumptive diagnosis; classic homoeopathy: the use of singular homeopathic remedies for individualised symptoms and patients), homotoxicology, traditional Chinese medicine (including herbal medicine and acupuncture), neural therapy, organotherapy, and Bach flower remedies [2–4, 10, 19–24]. In Germany, the term naturopathy is clearly separated from complementary treatment modalities [25, 26]. It includes phytotherapy, hirudotherapy, biophysical methods such as hydro-, thermo- and electrotherapy as well as manual therapies including chiropractic and osteopathy [8, 19, 21, 26]. Based on the challenge of closed definitions, some treatment options are mentioned in both categories [15–17, 27, 28], which is not critical for this study as there is no separation in the questionnaire itself.

In order to explore the current state of the field, this study aims to investigate which treatment modalities are used, their indications, and existing qualifications and information pathways among veterinarians. To map the current controversy about usage, evidence and study situation in this field, this study attempts to record the advantages and disadvantages of naturopathic and complementary treatments as experienced by veterinarians. As studies in human medicine show an influence of demographic data like gender and working environment for the use of complementary treatment modalities [12, 29, 30], demographic influences are investigated to determine their impact on applying veterinary naturopathy and complementary medicine.

## Material and methods

### Ethics and data protection

This study was approved by the Justus-Liebig-University Gießen (Germany) as part of a doctoral thesis, without the need for ethical approval. Before data collection, participants were informed of anonymity by an accompanying letter. By participating in the study, they consented to data collection. Tracking data back to individual persons was, and is, not possible in the data sets.

Each data set was labelled with continuous anonymous numbering. The accompanying letter gave background information, research contact data and assurance of anonymity (original cover letter available in S1 File).

### Data collection

An anonymous questionnaire was developed and distributed in both written and electronic form for this cross-sectional survey.

#### Target audience

The questionnaire targeted veterinary practitioners in small animal medicine throughout Germany (clients had to be at least 50% small animals). Based on the statistics of 2016 [31], 29,646 veterinarians of all specialisations were registered in Germany. Of these, 11,972 were self-employed (5,999 female), and 7,932 were employed (6,597 of them female). As represented in S1 Table, approximately 10,612 veterinarians were targeted with the inclusion criteria of this study [S1 Table]. More demographic data can be found in the official statistics of 2016 [31]. Due to data protection reasons, it is not possible to receive a full list of veterinarians from responsible authorities. Therefore, reaching the entire target group could not be an objective of this study. In order to determine an endpoint for collecting questionnaires, a comparison with another study was made. In their study about the use of veterinary phytotherapy in German-speaking countries (Germany, Austria, Switzerland), Hahn et al. [8] achieved a return rate of 7.8%. This return rate was used to orient the percentage of small animal veterinarians in Germany that were to be included in the study. It made the study more comparable with existing literature while also determining a feasible amount of questionnaires to be included in the study.

Based on the statistical data [31] and compared with the study of Hahn et al. [8], a cut-off of 10% for each German Chamber of Veterinary Surgeons was set. The targeted numbers of questionnaires for each Chamber of Veterinary Surgeons can be found in S1 Table. In total, it was set at 1061 questionnaires.

#### Questionnaire

Firstly, an extensive literature review was used to determine the methods of veterinary naturopathy and complementary medicine (using the databases of Pubmed, Livivo, and Google scholar as well as extensive manual research). Often mentioned treatment modalities in veterinary medicine were used for creating the questionnaire. A standardised questionnaire, with partly open fields for additional information, was designed (the questionnaire can be found in S1 and S2 Files in German and English). It used a verbalised rating scale without a mid-category and with an answer category similar to “kann ich nicht einschätzen / I cannot assess” to avoid bias for the items. For this type of rating scale, the participant had to choose between written answers in contrast to numeric scales or icons. The questionnaire was divided into five sections. The first section requested demographic data (age, gender, place of qualification, working field, other qualifications). The second section focused on using multiple-choice categories to apply veterinary naturopathy and complementary methods and fields in daily practice. Section three asked for a subjective impression of owner demand in the field of veterinary naturopathy and complementary medicine within the last five years. This timeline was based on the introduction of the qualification of “biological veterinary medicine” in the State Veterinary Association of Baden-Württemberg at the time of planning the questionnaire [32]. Section four investigated the information pathways used by veterinarians in naturopathy and complementary medicine and the assessment of this information. The last part of the questionnaire investigated the advantages and disadvantages that veterinary practitioners see in the application of these treatment modalities. The multiple-choice options were based on the literature review and were completed by an open field for individual additions. More than one response was possible for certain questions.

#### Distribution of questionnaire

After a trial with five participants in July and August of 2016, the advantage “work satisfaction” and the treatment option “Bach flower remedies” were added to the questionnaire. The final questionnaire was distributed from 01/09/2016 to 20/01/2018. The written questionnaire was distributed at several events in Germany personally or via open display (veterinarians were able to pick a questionnaire themselves) (S2 Table). An electronic form of the questionnaire was digitalised using Limesurvey Version 2016 and was available under http://vetmed.limequery.com/538872?lang=de. Requests for conducting the study were placed in the following German publications dedicated to veterinarians: “Tierärzteblatt”, “Tierärztliche Praxis” and “Informationsheft des bpt” 2017. Furthermore, social media was used to recruit participants for the study (Facebook). Appeals were posted every week in October 2017 in the private groups “Bund angestellter Tierärzte”, “Tierärzte”, “Diskussionsforum Veterinärmedizin” and “Tierärztinnen unter sich”.

### Data analysis

After data collection, all answers were summarised in a table (Microsoft Excel 2013 version 15.0). Multiple-choice categories were coded for clarity with a data set using numbers starting with zero upwards (in the case of questions 2, 3, 4.1, 5.1.1, 5.2.1). Other items were not coded, because of less categories. Afterwards, invalid data sets were erased (blanks from Limesurvey and those not meeting the inclusion criteria). Data sets with partly blank answers were used for further calculation, altering the statistical population for each item.

All answers to open questions (e. g. item 2.3 “other treatment modalities”) were combined as “other”. The open questions asking for treatment modalities with the most advantages or disadvantages (item 5.1.2 and 5.2.2) were categorised using the given treatment modalities of the questionnaire (2.3). All open questions were processed using descriptive statistics.

Statistical analysis was done using SAS 9.3. Metric data (item “age”) were described using median, minimum and maximum. All other items were described as categorical data, including “other” or “blank”. Answers were described in absolute and relative numbers. Relative numbers were based on the number of “not blank” answers as a statistical population.

Next, demographic data were analysed to calculate whether they influenced the use of veterinary naturopathy and complementary medicine by practitioners (user versus non-user). First, the Shapiro Wilk test tested metric data for normal distribution. The data were not normally distributed. Therefore, for metric data, the Mann-Whitney-U-Test was used. Because of the number of categories with categorical data, the exact Fisher-Test was applied. All analyses were performed as two-tailed tests.

## Results

### Survey response and description of participants

1087 questionnaires were received. The response rate cannot be calculated due to the distribution method, as described above. After applying the exclusion criteria, 870 data sets remained for evaluation. Table 1 shows the exclusion criteria and numbers of affected questionnaires. Compared to German veterinary statistics of 2016, 8% of all potentially includible veterinarians have been included in this study (S1 Table; [45]).

**Table 1:** Absolute numbers of included and excluded questionnaires (separated for exclusion criteria).

Participation	Included questionnaires [n /%]	Excluded questionnaires			
		Clients < 50 % Small animals	Completely blank data sets	Practicing abroad	Student
Online	427 / 49.1	46	110	0	0
Handwritten	443 / 50.9	44	2	14	1
Total	870 / 100	90	112	14	1

Women were represented with 716 (82.3%) questionnaires. The median age was 45 years with a minimum of 24 and a maximum of 76 years. S3 Table shows the distribution of the participants across the Chambers of Veterinary Surgeons. 97.6% (n = 849) of all participants named the university of final graduation (Table 2). While 94.8% graduated in Germany, 5.2% of the participants (n = 44) graduated in another country; these are listed in S4 Table separately.

**Table 2:** University of final graduation for participants*.

University of final graduation**	[n]	[%]
Gießen	215	25.3
Munich	212	25.0
Hannover	159	18.7
Berlin	141	16.6
Leipzig	78	9.2
Other answer	44	5.2
No answer	21	-

* Relative numbers calculated for a population of 849 (non-blank answers).

** In Germany five universities of veterinary medicine exist in Berlin, Gießen, Hannover, Leipzig, and Munich.

Self-employed veterinarians were represented with 62.7% (n = 544), employees with 33.3% (n = 289), “other” was named by 4% (n = 35), two participants did not answer this item. A little more than half of the veterinarians giving answers for this item worked in an urban environment (50.9%, n = 440), 47.5% (n = 411) worked in a rural environment, and 1.6% (n = 14) named both. When asked if they have “other educational achievements”, 71.6% (n = 619 of total 864) answered with “yes”, but only 245 of them stated a specific qualification. The named qualifications are listed in S5 Table.

### Implementation of veterinary naturopathy and complementary medicine

Out of all 861 given answers for this item 76.8% (n = 661) were interested in veterinary naturopathy and complementary medicine, 18.4% (n = 158) showed no interest, and 4.9% (n = 42) chose “no answer”. Users of related treatment modalities were 85.4% (n = 679). Complex homoeopathy and phytotherapy were cited most frequently (Table 3). The most frequently named indications were orthopaedic and geriatric issues, followed by metabolic diseases (Table 3).

**Table 3:** Use of veterinary naturopathy and complementary medicine sorted by medical fields*.

Treatment	Users [n,%]	Geriatrics [n]	Oncology [n]	Metabolic diseases [n]	Parasitic diseases [n]	Dermatology [n]	Infectious diseases [n]	Reproductve Medicine [n]	Behavioral diseases [n]	Orthopaedics [n]	Other [n]
Classic homoeopathy	301, 44.3%	198	145	182	33	169	157	149	165	188	5
Complex homoeopathy	478, 70.4%	353	258	322	38	259	291	211	201	351	10
Phyto-therapy	409, 60.2%	259	112	215	48	185	197	89	162	220	6
TCM**	205, 30.2%	108	49	77	20	77	55	58	60	166	6
Biophysical therapies	272, 40.1%	91	34	39	12	102	32	15	20	221	24
Manual therapies	240, 35.3%	106	14	25	8	15	11	20	37	212	5
Diverting therapies	149, 21.9%	39	7	24	7	43	20	9	11	114	6
Bach flower remedies	249, 36.7%	48	21	27	13	27	20	24	228	33	0
Neural therapy	134, 19.7%	35	8	15	5	12	9	17	11	117	2
Homo-toxicology	108, 15.9%	84	77	84	17	76	73	59	53	80	2
Organo-therapy	135, 19.9%	91	78	98	19	68	56	49	49	81	3
Other	53, 7.8%	16	16	16	11	17	15	10	15	15	1
Total	-	1428	819	1124	231	1050	936	710	1012	1798	70

* Multiple choices possible, relative numbers calculated for a population of users of treatment and for 679 users in total.

** Traditional Chinese medicine.

### Customer demand

Increasing demand for veterinary naturopathy and complementary medicine over the past five years was stated by 57.9% (n = 457 of total 798) of participants. A stable demand was stated by 28.5% (n = 225), 2.7% (n = 21) estimated a decreasing demand, and 10.9% (n = 21) could not estimate the development of demand.

### Information pathways

Table 4 shows the absolute and relative numbers of information pathways used by veterinarians. Scientific literature and books were used most often. The information was rated as adequate by 16.7% (n = 130 of total 779), 22.8% (n = 178) rated it nearly adequate, 25.9% (n = 202) nearly not adequate, and 6.8% (n = 53) as not adequate. The information could not be evaluated by 27.7% (n = 216) of the participants.

**Table 4:** Information pathways, advantages, and disadvantages in the use of veterinary naturopathy and complementary medicine named by veterinarians*.

Information pathways	[n]	[%]
Scientific literature	479	60.8
ATF**-certified further education	383	48.6
Company information	351	44.5
Colleagues	333	42.3
Internet	316	40.1
Further veterinary education	185	23.5
Further not-veterinary education	121	15.4
Contents in university / studies	29	3.7
Other	41	5.2
None / not interested	105	13.3
Blank answer ***	82	-
**Disadvantages**		
Ambiguous study situation / unclear evidence for mode of action and effectiveness	483	62.1
Owner anticipation	393	50.5
Qualitative blank information	244	31.4
Lack of time	235	30.2
Quantitative blank information	122	15.7
No disadvantages	82	10.5
Other	79	10.2
Interdependancy with other treatments	71	9.1
Blank answer ***	92	-
**Advantages**		
Expansion of treatment options	566	73.5
Higher customer satisfaction	545	70.8
Treatments with less side effects	487	63.2
Higher job satisfaction	364	47.3
Extended monetary potential	236	30.6
No advantages	59	7.7
Other	40	5.2
Blank answer ***	100	-

* Multiple choices possible, relative numbers calculated for a population of non-blank answers.

**ATF: Academy for Veterinary Continuing Education, an organisation that certifies independent veterinary continuing education in Germany.

*** [n] of 870 questionnaires; [%] not calculated, as given percentages in the table refer to the population of respondents for each item.

### Advantages and disadvantages in the use of naturopathy and complementary medicine as seen by veterinarians

The most frequently cited disadvantage was “ambiguous study situation / unclear evidence for the mode of action and effectiveness” (Table 4). Table 4 shows absolute and relative numbers of other disadvantages. When asked for which treatment option these disadvantages are seen the most, homoeopathic treatments were named most often (S6 Table).

A wider variety of treatment options was the most frequently named advantage of veterinary naturopathy and complementary medicine, followed by more satisfied customers. The incidence of advantages is shown in Table 4. Manual therapies like chiropractic were seen as the treatment option with the most advantages (49.9%, n = 227 of total 455). S7 Table shows an overview of rated advantages for other treatment options.

### Influence of demographic parameters on the use of veterinary naturopathy and complementary medicine

Age, gender, and mode of employment show a statistically significant impact on the use of veterinary naturopathy and complementary medicine by the participants (p < 0.001) (Tables 5 and 6). Users of veterinary naturopathy and complementary medicine tend to be older, female and self-employed. With the used statistical test, the place of final examination also showed a statistical impact (p < 0.027), although it seems to be weaker than the demographic factors named above (Table 6). Those who graduated from the University of Berlin were more likely to use these treatment modes.

**Table 5:** Age and its impact on the use of veterinary naturopathy and complementary medicine (Mann-Whitney-U-Test)*.

Variable	Usage	[n]	25th Percentil	Median	75th Percentil	Max.	p
**Age [Years]**	no	115	34.0	41.0	51.0	76.0	<0.001
	yes	676	38.0	47.0	53.5	74.0	

*based on non-blank answers.

**Table 6:** Non-metric demographic data and their impact on the use of veterinary naturopathy and complementary medicine (Exact Fischer-Test)*.

Item	Answer	Usage				p
		no		yes		
		[n]	[%]	[n]	[%]	
**Gender**	Male	36	24.5	111	75.5	< 0.001
	Female	80	12.3	569	87.7	
**University of final graduation**	Berlin	12	9.2	119	90.8	0.027
	Gießen	23	11.7	174	88.3	
	Hannover	30	22.2	105	77.8	
	Leipzig	10	14.1	61	85.9	
	Munich	32	15.8	170	84.2	
**Work environment (urban / rural)**	Rural	46	12.0	336	88.0	0.054
	Urban	67	17.0	328	83.0	
**Mode of employment**	Employee	55	22.0	195	78.0	< 0.001
	Self-employed	58	11.4	453	88.6	
**Further education**	Yes	31	13.5	199	86.5	0.657
	No	84	15.0	477	85.0	

* based on non-blank answers of each category.

## Discussion

International authors have stated an increasing interest in veterinary naturopathy and complementary medicine [1–5]. This study aimed to describe the use of naturopathy and complementary medicine in Germany by veterinarians in small animal medicine for the first time.

To answer the initial questions, a cross-sectional study using a questionnaire is a feasible option. It allows participants to answer anonymously and enables the collection of large numbers of data in a short period of time; however, it has to simplify information [33, 34]. This questionnaire reduces information and summarises different treatment options (regarding the level of evidence and opinion of medicine or disease). Furthermore, the possibility of open answers in questionnaires can be detrimental to some extent [33, 34]. In particular, there is a loss of information regarding the item “further education”, due to many imprecise answers. For an answer to be included, it was necessary to name the precise specialisation only. For example, naming a field of interest like “homoeopathy” could not be evaluated. Stating the treatment option, where the advantages or disadvantages were most prevalent, was another open question. A lack of understanding of the questionnaire is assumed here. Many participants named the advantages or disadvantages instead of the treatment modality, leading to lower numbers of usable data sets, as those were not included in the sample size for this item.

Consistent with German veterinary statistics of 2016, about 8% of the targeted population has been polled (S1 Table; [45]). A majority (85.4%) of participants used veterinary naturopathy and complementary treatment options in their practical work. This is a high response rate, even though it is not possible to achieve full representation due to the voluntary nature of the questionnaire and its distribution. Although avoidance of bias was attempted by using different distribution channels via Internet, veterinary journals, display, or personal hand-outs, participation remained voluntary. The inclusion of veterinarians particularly interested in this topic, is a bias that cannot be excluded and presents a restriction in interpreting the data. On the other hand, 14.6% of participants did not use these treatment options, showing that not only practitioners of these methods participated in the study.

Comparing the data is challenging because of the limited available data which describes the existing use of veterinary naturopathy and complementary medicine. In summary, with 85.4%, the use of veterinary naturopathy and complementary medicine in the present study was higher than in most previous studies in human and veterinary medicine [6–9, 11–13], except that of Alscher [12]. These high numbers may be due to an existing bias, as mentioned above. Additionally, there could be a greater pet owner interest in these treatment modalities, but data exploring this are scarce. Most previous studies have the same bias because of study design and voluntary participation. Because of the retrospective design of the study of Shmalberg and Memon [9], this bias is avoided. They show comparable high numbers, however, comparability is limited as homoeopathy or chiropractic treatments were not included in the study [9].

The most commonly used treatment option in this study is homoeopathy. Simultaneously, homoeopathy was ranked as the treatment option with the most disadvantages. This high percentage could be due to owner demand, personal experience using homoeopathy, monetary concerns, and the current ongoing public discussion. The data cannot offer distinct motivation here. Phytotherapy was the second most used treatment option in this study. Less frequently named treatment options were homotoxicology, neural therapy, and organotherapy. Lana et al. showed that homoeopathy, phytotherapy, acupuncture, chiropractic and Bach flower remedies are used by owners treating oncologic diseases in their pets [10]. Without providing numbers, Arlt and Heuwieser [1] also state that acupuncture, phytotherapy, and homoeopathy are frequently used techniques in veterinary medicine. Shmalberg and Memon showed 81.5% use of acupuncture for their patients [9]. It must be noted that although manual therapies and TCM (Traditional Chinese medicine) are named as the treatments with the highest advantages, these are not found in the most frequently used treatments. Based on the questionnaire result, it cannot be deciphered whether this is due to owner demand, a requirement of further education, or even monetary reasons. Overall, it can be assumed that owners seek the same treatment options for their pets as they would use for themselves since the treatment options that are mainly used among the German people are comparable [35, 36].

Most often, homoeopathy is used for geriatric patients, followed by orthopaedics. It is also frequently cited for the other treatment fields except for parasitic diseases. In a retrospective study about chronic diseases and homoeopathy in small animal medicine, Mathie et al. [37] similarly showed orthopaedic problems as a common field where complementary medicines were used. The large numbers of orthoapedic application could be due to existing complex homoeopathic remedies and their publicity in German veterinary and human medicine. Furthermore, orthopaedic problems like osteoarthrosis are a part of chronic diseases and palliative medicine. In a review by Hektoen [38], chronic diseases and palliative medicine are mentioned as special fields of homoeopathy. It is assumed that geriatric patients show chronic diseases and palliative situations more frequently and therefore amount to a larger portion of this group. However, Clausen et al. [39] state there is limited evidence-based information available as only a few studies are available and exist only for dogs in the fields of dermatology, behavioural diseases, and infectious diseases.

To summarise all diseases where naturopathic or complementary medicine is used, orthopaedics, geriatrics, metabolic diseases, and dermatology appear most often. These fields are similarly mentioned for phytotherapy by Hahn et al. [8], for acupuncture by Habacher et al. [40], and for homoeopathy by Mathie et al. [37].

The most consistently used information pathways for veterinarians in the field of naturopathy and complementary medicine in this study were scientific journals and books. There was no item asking for a definition by the participants of what “scientific” meant for them. Therefore, scientific levels could be diverse. Information from primary academic education was less frequently cited. Memon et al. [41] suggest content for a possible veterinary student curriculum. They pointed out how important the content is for veterinarians to assess treatment options critically [41]. This could be of great interest as Lana et al. [10] stated that 46% of owners use veterinarians for information about complementary veterinary medicine. The overall, more positively assessed, information coincides with relatively few participants who deemed the lack of information as a disadvantage. This conflicts with authors who write that evidence-based literature for veterinary naturopathy and complementary medicine is relatively rare [2, 42] except for acupuncture [1, 40].

This study could not fully answer the question of veterinary users’ existing qualifications due to the open character of the question mentioned above. A rather diverse field of qualifications was found, these are listed in S5 Table. No significant influence of further education on the use of naturopathic or complementary medicine was found. The study of Truls [6] showed that most veterinary practitioners did not participate in specialised (phytotherapeutic) further education. In addition to this, authors like Milstein state that a qualification does not provide evidence for the effectiveness of the treatment [43].

The most commonly named advantages of veterinary naturopathy and complementary medicine were more extensive treatment options, followed by more satisfied customers. The third advantage of fewer side effects is mentioned by other authors too [5, 8, 44–47]. Nevertheless, these treatment options have their side effects [2, 5, 48, 49]. Users of these treatment options should be aware of the side effects, which means having knowledge of the side effects, interactions with other treatments, treatment limits, and application of the treatment itself. The higher work satisfaction for veterinarians using naturopathy and complementary medicine is paralleled by human studies [4, 11].

Only 10.5% of respondents see no disadvantages in using veterinary naturopathy and complementary medicine. The lack of evidence and knowledge of the mode of action is considered the primary disadvantage. This reflects data in the existing literature [2, 5, 1, 39]. For the use of evidence-based medicine, there is a need for well-designed studies to acquire more valid information in this field [1, 40, 42, 50]. Acupuncture especially shows a growing database of well-designed studies [51–54]. In addition, the participants of this study mentioned not having time for veterinary naturopathy and complementary medicine. This might be due to the often detailed and time-consuming owner anamnesis and the use of different diagnostic approaches, especially in homoeopathy and TCM [3, 4, 37, 55].

Gender, graduating university, and type of employment showed a significant impact on the use of veterinary naturopathy and complementary medicine (Table 6). A survey from Härtel and Volger [36] about naturopathy and alternative treatments among the general German human population revealed that 70% of the female participants versus 54% of the male participants were using these techniques. For US-American pet owners, Lana et al. [10] showed that of 254 owners, 76% applied veterinary complementary and alternative treatments to combat oncologic diseases. A majority were female, showing a statistically significant impact of p = 0.003 for gender. The more frequent use of (veterinary) naturopathy and complementary medicine by females seems to originate in social concepts of caretaking as well as a less mechanistic view of healing and disease [30].

Although the data shows a statistical impact of graduating from different universities on the use of veterinary naturopathy and complementary medicine, it is weaker than the impact made by other factors. A multiple-comparison post-hoc correction test might result in different findings. It is assumed that the contents of naturopathy and complementary medicine in the curricula could influence the adoption of these modalities. However, there is no available data on the curricula of the different German universities in this field and their development over the last decades. In some German universities, these topics are already part of the elective courses, as is the case at the University of Berlin. The slightly higher percentage of veterinaries who use naturopathic or complementary treatments may be due to the existing scientific research group for naturopathy and complementary subjects at the University of Berlin.

The statistical influence of the type of employment could overlap with the age factor. It is assumed that self-employed veterinarians tend to be older than employed ones. Furthermore, self-employed veterinarians tend to have a greater scope for choosing their treatment options since the owner of the clinic or practice might influence the available treatment options.

No statistical influence was found for practitioners working in a rural or urban environment. However, Alscher [12] showed a statistically significant correlation between rural or urban working environments and the adoption of naturopathic or complementary therapies in human medicine. With the development of veterinary medicine infrastructure (e. g. in terms of digitalisation and diagnostic possibilities), it seems reasonable for the rural and urban medical supplies to equalise.

## Conclusions

Despite a bias due to voluntary participation, this study shows widespread use of naturopathy and complementary small animal medicine in Germany. Nevertheless, uncertainties in evidence, safety and, qualification for use exist. Homoeopathy represents this uncertainty as it is cited as the most frequently used treatment option despite being seen as the treatment with the most potential disadvantages. The present study, with its restrictions, supports the need for a discussion about evidence, official regulations, and the need for official qualifications due to the widespread application of veterinary naturopathy and complementary medicine. More data about the modes of action and effectiveness of modalities are needed in order to enable veterinarians to provide evidence-based advice to pet owners.

## Supporting information

S1 Table Calculation of distribution questionnaire. (DOCX)

S2 Table Events used for distribution. (DOCX)

S3 Table Distribution questionnaires in Germany (State Veterinary Association). (DOCX)

S4 Table Foreign graduation universities. (DOCX)

S5 Table Frequently named qualifications of participants. (DOCX)

S6 Table Treatments with potential disadvantages. (DOCX)

S7 Table Treatments with potential advantages. (DOCX)

S1 File German questionnaire. (PDF)

S2 File English questionnaire. (PDF)

S1 Dataset Main data set. English.
(XLSX)

S2 Dataset Main data set. (XLSX)

## References

[1] Arlt S, Heuwieser W. Evidence-Based Complementary and Alternative Veterinary Medicine—a contradiction in terms? Berl Munch Tierarztl Wochenschr. 2010;123(9–10):377–84. doi: 10.2376/0005-9366-123-377 21038809

[2] Raditic DM. Complementary and integrative therapies for lower urinary tract diseases. Vet Clin North Am Small Anim Pract. 2015;45(4):857–78. doi: 10.1016/j.cvsm.2015.02.009 25838155

[3] Pesch L. Holistic pediatric veterinary medicine. Vet Clin North Am Small Anim Pract. 2014;44(2):355–66. doi: 10.1016/j.cvsm.2013.11.003 24580996 PMC7132486

[4] Kidd JR. Alternative medicines for the geriatric veterinary patient. Vet Clin North Am Small Anim Pract. 2012;42(2):809–22. doi: 10.1016/j.cvsm.2012.04.009 22720815

[5] Budgin JB, Flaherty MJ. Alternative therapies in veterinary dermatology. Vet Clin North Am Small Anim Pract. 2013;43(1):189–204. doi: 10.1016/j.cvsm.2012.09.002 23182332

[6] Truls C. Der Einsatz von pflanzlichen Arzneien in der Kleintierpraxis [dissertation] Vienna: Veterinary University Vienna; 1999.

[7] Ertl M. Der Einsatz von pflanzlichen Arzneimitteln in Tierarztpraxen in Kärnten. [dissertation] Vienna: Veterinary University Vienna; 2002.

[8] Hahn I, Zitterl-Eglseer K, Franz C. Phytomedizin bei Hund und Katze: Internetumfrage bei Tierärzten und Tierärztinnen in Osterreich, Deutschland und der Schweiz. Schweiz Arch Tierheilkd. 2005;147(3):135–41. doi: 10.1024/0036-7281.147.3.135 15801625

[9] Shmalberg J, Memon MA. A Retrospective Analysis of 5,195 Patient Treatment Sessions in an Integrative Veterinary Medicine Service: Patient Characteristics, Presenting Complaints, and Therapeutic Interventions. Vet Med Int. 2015;1:1–11. doi: 10.1155/2015/983621 26798552 PMC4699059

[10] Lana SE, Kogan LR, Crump KA, Graham JT, Robinson NG. The use of complementary and alternative therapies in dogs and cats with cancer. J Am Anim Hosp Assoc. 2006;42(5):361–5. doi: 10.5326/0420361 16960039

[11] Baatsch B, Zimmer S, Rodrigues RD, Büssing A. Complementary and alternative therapies in dentistry and characteristics of dentists who recommend them. Complement Ther Med. 2017;35:64–9. doi: 10.1016/j.ctim.2017.08.008 29154070

[12] Alscher AC. Verwendung und Bewertung von komplementären Verfahren unter niedergelassenen Allgemeinmedizinern, Internisten und Orthopäden. Ergebnisse einer bundesweiten Befragung [dissertation]. Munich: Technical University Munich; 2018.

[13] Thanner M, Nagel E, Loss J. Komplementäre und alternative Heilverfahren im vertragsärztlichen Bereich: Ausmaß, Struktur und Gründe des ärztlichen Angebots. Gesundheitswesen. 2014;76(11):715–21. doi: 10.1055/s-0033-1364013 24566840

[14] Walach H, Pietikäinen S. A roadmap for CAM research towards the horizon of 2020. Forsch Komplementmed. 2014;21(2):80–1. doi: 10.1159/000358105 24851841

[15] Holmberg C, Brinkhaus B, Witt C. Experts’ opinions on terminology for complementary and integrative medicine—a qualitative study with leading experts. BMC Complement Altern Med. 2012;12:218–24. doi: 10.1186/1472-6882-12-218 23151006 PMC3522550

[16] Gaboury I, April KT, Verhoef M. A qualitative study on the term CAM: Is there a need to reinvent the wheel? BMC Complement Altern Med. 2012;12:131. doi: 10.1186/1472-6882-12-131 22909051 PMC3462712

[17] Wieland LS, Manheimer E, Berman BM. Development and classification of an operational definition of complementary and alternative medicine for the Cochrane collaboration. Altern Ther Health Med. 2011;17(2):50–9. 21717826 PMC3196853

[18] Weltgesundheitsorganisation. WHO traditional medicine strategy 2014–2023. Genève: WHO; 2013.

[19] Kübler H, Brendieck-Worm C, editors. Naturheilkundliche Therapieverfahren: Information brochure of the GGTM e.V. for pet owners. Schallstadt: GGTM e.V; 2015.

[20] Kline KL. Complementary and alternative medicine for neurologic disorders. Clin Tech Small Anim Pract. 2002;17(1):25–33. doi: 10.1053/svms.2002.29075 11890124

[21] Stock-Schröer B, Lieverscheidt H, Frei-Erb M. Curriculum Naturheilverfahren und Komplementärmedizin: Lehrinhalte und Medizindidaktik. Essen: KVC; 2013.

[22] Berschneider HM. Complementary and alternative veterinary medicine and gastrointestinal disease. Clin Tech Small Anim Pract. 2002;17(1):19–24. doi: 10.1053/svms.2002.30224 11890123

[23] Hawks D. Alternative medicine: musculoskeletal system. Clin Tech Small Anim Pract. 2002;17(1):41–9. doi: 10.1053/svms.2002.27784 11890127

[24] Buffington CA. Complementary and alternative veterinary medicine and urologic conditions. Clin Tech Small Anim Pract. 2002;17(1):34–6. doi: 10.1053/svms.2002.27059 11890125

[25] Hufelandgesellschaft e. V. Klassische Naturheilkunde. 2020. http://www.hufelandgesellschaft.de/klassische_naturheilkunde.html. Accessed 31 Aug 2020.

[26] Kraft K, Stange R. Lehrbuch Naturheilverfahren. 1st ed. Stuttgart: Hippokrates; 2010.

[27] Fischer F, Lewith G, Witt CM, Linde K, Ammon Kv, Cardini F, et al. A research roadmap for complementary and alternative medicine—what we need to know by 2020. Forsch Komplementmed. 2014;21(2):e1–16. doi: 10.1159/000360744 24851850

[28] Cassidy CM. Commentary on terminology and therapeutic principles: Challenges in classifying complementary and alternative medicine practices. J Altern Complement Med. 2002;8(6):893–5. doi: 10.1089/10755530260511883 12619650

[29] Linde K, Alscher A, Friedrichs C, Joos S., Schneider A. Die Verwendung von Naturheilverfahren, komplementären und alternativen Therapien in Deutschland—eine systematische Übersicht bundesweiter Erhebungen. Forsch Komplementmed. 2014;21(2):111–8. doi: 10.1159/000360917 24851848

[30] Zhang Y, Leach MJ, Hall H, Sundberg T, Ward L, Sibbritt D, et al. Differences between Male and Female Consumers of Complementary and Alternative Medicine in a National US Population: A Secondary Analysis of 2012 NIHS Data. Evid Based Complement Alternat Med. 2015; epub2015:413173. doi: 10.1155/2015/413173 25861360 PMC4377351

[31] Bundestierärztekammer. Statistik 2016: Tierärzteschaft in der Bundesrepublik Deutschland. Deutsches Tierärzteblatt. 2017:616–21.

[32] Bundestierärztekammer. Zusatzbezeichnung Biologische Tiermedizin. 2019. https://www.bundestieraerztekammer.de/btk/musterordnungen/weiterbildung/. Accessed 31 Aug 2020.

[33] Thielsch MT, Weltzin S. Online-Befragungen in der Praxis. In: Brandenburg T, Thielsch M., T., editors. Praxis der Wirtschaftspsychologie: Themen und Fallbeispiele für Studium und Anwendung. Münster: MV Wissenschaft; 2009. p. 69–85.

[34] Moosbrugger H, Kelava A, editors. Testtheorie und Fragebogenkonstruktion: Mit 66 Abbildungen und 41 Tabellen. 2nd ed. Berlin, Heidelberg: Springer; 2012. doi: 10.1186/1742-9994-9-29

[35] Bücker B, Groenewold M, Schoefer Y, Schäfer T. The use of complementary alternative medicine (CAM) in 1001 German adults: Results of a population-based telephone survey. Gesundheitswesen. 2008;70(8–9):29–36. doi: 10.1055/s-2008-1081505 18785094

[36] Härtel U, Volger E. Inanspruchnahme und Akzeptanz klassischer Naturheilverfahren und alternativer Heilmethoden in Deutschland—Ergebnisse einer reprasentativen Bevolkerungsstudie. Forsch Komplementmed Klass Naturheilkd. 2004;11(6):327–34. doi: 10.1159/000082814 15604623

[37] Mathie RT, Baitson ES, Hansen L, Elliott MF, Hoare J. Homeopathic prescribing for chronic conditions in feline and canine veterinary practice. Homoeopathy. 2010;99(4):243–8. doi: 10.1016/j.homp.2010.05.010 20970093

[38] Hektoen L. Review of the current involvement of homoeopathy in veterinary practice and research. Vet Rec. 2005;157(8):224–9. doi: 10.1136/vr.157.8.224 16113167

[39] Clausen J, Albrecht H, Mathie RT. Veterinary clinical research database for homoeopathy: placebo-controlled trials. Complement Ther Med. 2013;21(2):115–20. doi: 10.1016/j.ctim.2012.11.009 23497815

[40] Habacher G, Pittler MH, Ernst E. Effectiveness of Acupuncture in Veterinary Medicine: Systematic Review. J Vet Intern Med. 2006;20(3):480–8. doi: 10.1892/0891-6640(2006)20[480:eoaivm]2.0.co;2 16734078

[41] Memon MA, Shmalberg J, Adair HS III, Allweiler S, Bryan JN, Cantwell S, et al. Integrative veterinary medical education and consensus guidelines for an integrative veterinary medicine curriculum within veterinary colleges. Open Vet J. 2016;6(1):44–56. doi: 10.4314/ovj.v6i1.7 27200270 PMC4824037

[42] McKenzie BA. Is complementary and alternative medicine compatible with evidence-based medicine? J Am Vet Med Assoc. 2012;241(4):421–6. doi: 10.2460/javma.241.4.421 22852564

[43] Milstein. The case against alternative medicine. Can Vet J. 2000;41(10)769–72. 11062833 PMC1476381

[44] Vockeroth WG. Veterinary homoeopathy: An overview. Can Vet J. 1999;40(8):592–4. 12001344 PMC1539764

[45] Wynn SG, Chalmers S. Alternative therapies for pruritic skin disorders. Clin Tech Small Anim Pract. 2002;17(1):37–40. doi: 10.1053/svms.2002.27058 11890126

[46] Bravo-Monsalvo A, Vazquez-Chagoyan JC, Gutierrez L, Sumano H. Clinical efficacy of neural therapy for the treatment of atopic dermatitis in dogs. Acta Vet Hung. 2008;56(4):459–69. doi: 10.1556/AVet.56.2008.4.4 19149101

[47] Luna SPL, Di Martino I, Lorena S. E. R de Sá, Capua M. L. B. de, Lima AF, Santos B. P. C. R. dos, et al. Acupuncture and pharmacopuncture are as effective as morphine or carprofen for postoperative analgesia in bitches undergoing ovariohysterectomy. Acta Cir Bras. 2015;30(12):831–7. doi: 10.1590/S0102-865020150120000007 26735055

[48] Poppenga RH. Herbal medicine: potential for intoxication and interactions with conventional drugs. Clin Tech Small Anim Pract. 2002;17(1):6–18. doi: 10.1053/svms.2002.27785 11890130

[49] Daniels M, Bartges JW, Raditic DM, Marsden S., Cox SK, Callens AJ. Evaluation of three herbal compounds used for the management of lower urinary tract disease in healthy cats: A pilot study. J Feline Med Surg. 2018;20(12):1094–9. doi: 10.1177/1098612X17748241 29256321 PMC11104217

[50] Overall KL, Dunham AE. Homoeopathy and the curse of the scientific method. Vet J. 2009;180(2):141–8. doi: 10.1016/j.tvjl.2008.10.006 19046906

[51] Lane DM, Hill SA. Effectiveness of combined acupuncture and manual therapy relative to no treatment for canine musculoskeletal pain. Can Vet J. 2016;57(4):407–14. 27041759 PMC4790233

[52] Liu CM, Chang FC, Lin CT. Retrospective study of the clinical effects of acupuncture on cervical neurological diseases in dogs. J Vet Sci. 2016;17(3):337–45. doi: 10.4142/jvs.2016.17.3.337 26645331 PMC5037301

[53] Scallan EM, Simon BT. The effects of acupuncture point Pericardium 6 on hydromorphone-induced nausea and vomiting in healthy dogs. Vet Anaesth Analg. 2016;43(5):495–501. doi: 10.1111/vaa.12347 26890432

[54] Roynard P, Frank L, Xie H, Fowler M. Acupuncture for Small Animal Neurologic Disorders. Vet Clin North Am Small Anim Pract. 2018; 48(1):201–19. doi: 10.1016/j.cvsm.2017.08.003 29037432

[55] Cantwell SL. Traditional Chinese veterinary medicine: the mechanism and management of acupuncture for chronic pain. Top Companion Anim Med. 2010;25(1):53–8. doi: 10.1053/j.tcam.2009.10.006 20188339

