# Supplementary material for: Application of veterinary naturopathy and complementary medicine in small animal medicine—A survey among German veterinary practitioners
Source: PLoS One. 2022 Feb 28;17(2):e0264022. doi: 10.1371/journal.pone.0264022 (PMC8884514; doi:10.1371/journal.pone.0264022)
Supplement: S1 Table — (DOCX) [file pone.0264022.s001.docx]

| **Chamber of Veterinary Surgeons of:** | Self-employed (small animal science)(A) | Total of self-employed (B) | C (%)  = A/B | Total of employees (D) | Assessment employees (small animal)  E = C * D | Total debuty vet.  (F) | Assessment debuty vet. (small animal)  G = C * F | **Sum (5 %)** = (A+E+G)*0,05 | **Sum (10 %)**= (A+E+G)*0,1 |
| --- | --- | --- | --- | --- | --- | --- | --- | --- | --- |
| Baden- Württemberg | 566 | 1193 | 0.47 | 786 | 372.90 | 22 | 10.43 | 47.46 | 94.93 |
| Bavaria | 1153 | 2284 | 0.50 | 1460 | 737.03 | 116 | 58.56 | 97.43 | 194.86 |
| Berlin | 377 | 387 | 0.97 | 201 | 195.81 | 29 | 28.25 | 30.05 | 60.11 |
| Brandenburg | 216 | 541 | 0.4 | 280 | 111.79 | 14 | 5.59 | 16.67 | 33.34 |
| Bremen | 48 | 53 | 0.91 | 37 | 33.51 | 2 | 1.81 | 4.17 | 8.33 |
| Hamburg | 148 | 170 | 0.87 | 86 | 74.87 | 5 | 4.35 | 11.36 | 22.72 |
| Hessen | 581 | 972 | 0.6 | 558 | 333.54 | 33 | 19.72 | 46.71 | 93.43 |
| Mecklenburg- Vorpommern | 64 | 273 | 0.23 | 1235 | 289.52 | 68 | 15.94 | 18.47 | 36.95 |
| Niedersachsen | 695 | 1614 | 0.43 | 983 | 423.29 | 33 | 14.21 | 56.63 | 113.25 |
| Nordrhein | 763 | 1109 | 0.69 | 835 | 574.49 | 33 | 22.70 | 68.01 | 136.02 |
| Westfalen-Lippe | 506 | 954 | 0.53 | 835 | 442.88 | 36 | 19.09 | 48.4 | 96.8 |
| Rheinland-Pfalz | 277 | 537 | 0.52 | 352 | 181.57 | 6 | 3.1 | 23.08 | 46.17 |
| Saarland | 74 | 123 | 0.60 | 98 | 58.96 | 1 | 0.60 | 6.68 | 13.36 |
| Saxony | 184 | 536 | 0.34 | 275 | 94.40 | 5 | 1.72 | 14.01 | 28.01 |
| Sachsen-Anhalt | 132 | 323 | 0.41 | 159 | 64.98 | 1 | 0.41 | 9.87 | 19.74 |
| Schleswig-Holstein | 254 | 591 | 0.43 | 470 | 201.1 | 13 | 5.59 | 23.08 | 46.16 |
| Thüringen | 119 | 312 | 0.38 | 133 | 50.73 | 1 | 0.38 | 8.51 | 17.01 |
| **Total** |  |  |  |  |  |  |  | **530**.59 | **1061**.17 |

**S1 Table: Calculation of total number and distribution of questionnaires (Based on statistical veterinary data of 2016 [31])**
