# Supplementary material for: Application of veterinary naturopathy and complementary medicine in small animal medicine—A survey among German veterinary practitioners
Source: PLoS One. 2022 Feb 28;17(2):e0264022. doi: 10.1371/journal.pone.0264022 (PMC8884514; doi:10.1371/journal.pone.0264022)
Supplement: S2 Table — (DOCX) [file pone.0264022.s002.docx]

**S2 Table: Events used for distribution of the questionnaire.**

- 30.09.2016-01.10.2016 Ultrasonografie Abdomen Intensive II, Tuttlingen
- 28.01.2017 Seminar for Internal Medicine, Ludwigsburg
- 16.02.2017 Congress for Small Animal Science, Baden Baden
- 21.09.2017-23.09.2017 „Kleintier auf der Insel Rügen“, Rügen
- 27.09.2017 „Gelenkspezifische Behandlung von Arthropathien mit biologischen Arzneimitteln“, Rostock
- 30.09.2017-01.10.2017 „Regulationsmedizin-Erkrankungen des Bewegungsapparates“, Timmendorfer Strand
- 07.10.2017 Further education for veterinary pharmacy and usage (bpt)
- 14.10.2017 „Möglichkeiten der biologischen Behandlung bei chronisch kranken Kleintierpatienten“, Berlin
- 19.10.2017-21.10.2017 bpt-Congress München
- 28.10.2017 „Wissen vor Ort – Sport- und Freizeitverletzungen im Fokus. Besonderheit bei der Behandlung von Hunden“, Frankfurt a. M.
- 18.11.2017 „Anorexie beim Kleinsäuger“, Anicura Heilbronn
- 22.11.2017 Seminar for Surgical Techniques, Veterinary hospital Ludwigsburg-Ossweil
- 09.12.2017-10.12.2017 PetVet Karlsruhe
