# Supplementary material for: Application of veterinary naturopathy and complementary medicine in small animal medicine—A survey among German veterinary practitioners
Source: PLoS One. 2022 Feb 28;17(2):e0264022. doi: 10.1371/journal.pone.0264022 (PMC8884514; doi:10.1371/journal.pone.0264022)
Supplement: S3 Table — (DOCX) [file pone.0264022.s003.docx]

| **Chamber of Veterinary Surgeons of** | **Absolut number of participants [n]** | **Relative number of participants [%]** |
| --- | --- | --- |
| Baden-Württemberg | 200 | 23.3 |
| Bayern / Bavaria | 141 | 16.4 |
| Berlin | 46 | 5.3 |
| Brandenburg | 28 | 3.3 |
| Bremen | 3 | 0.3 |
| Hamburg | 8 | 0.9 |
| Hessen | 84 | 9.8 |
| Mecklenburg-Vorpommern | 11 | 1.3 |
| Niedersachsen | 80 | 9.3 |
| Nordrhein | 67 | 7.8 |
| Westfalen-Lippe | 40 | 4.7 |
| Rheinland-Pfalz | 59 | 6.9 |
| Saarland | 10 | 1.2 |
| Sachsen / Saxony | 26 | 3.0 |
| Sachsen-Anhalt | 18 | 2.1 |
| Schleswig-Holstein | 24 | 2.8 |
| Thüringen | 13 | 1.5 |
| Fehlende Angabe | 10 | - |
| Angabe von zwei Kammern | 2 | 0.1 |
| Gesamtanzahl / Total | 870 | - |

**S3 Table: Distribution of participants listed for Chambers of Veterinary Surgeons in absolute and relative numbers (relative numbers calculated for main unit of 870 included questionnaires)**
