# Supplementary material for: Application of veterinary naturopathy and complementary medicine in small animal medicine—A survey among German veterinary practitioners
Source: PLoS One. 2022 Feb 28;17(2):e0264022. doi: 10.1371/journal.pone.0264022 (PMC8884514; doi:10.1371/journal.pone.0264022)
Supplement: S4 Table — (DOCX) [file pone.0264022.s004.docx]

| **Country / University of final examination** | **Absolut number of participants [n]** |
| --- | --- |
| Belgium (Ghent University) | 2 |
| Bulgaria (Trakia University, Stara Zagora) | 1 |
| Denmark (University of Copenhagen) | 1 |
| Italy | 5 |
| Università degli Studi di Milano | 1 |
| Università degli Studi di Parma | 1 |
| Università degli Studi di Perugia | 3 |
| Netherlands (Utrecht University) | 1 |
| Austria (Veterinary University of Wien) | 13 |
| Poland | 4 |
| [Warmia & Mazury University Olsztyn](https://hochschulen.studiumineuropa.eu/s/3542/74990-Deutsch-version/434-Warmia-und-Mazury-Universitat-in-Olsztyn.htm?muz=m) | 2 |
| Medical University Breslau | 2 |
| Romania (Banat University Of Agricultural Sciences And Veterinary Medicine Timisoara) | 1 |
| Spain (Universitat Autònoma de Barcelona) | 1 |
| Switzerland | 2 |
| Vetsuisse – Faculty Bern | 1 |
| Vetsuisse – Faculty Zürich | 1 |
| Czech Republic (Veterinàrnì a Farmaceutickà Univerzita Brno) | 2 |
| Ukraine (University for Animal Breeding and Veterinary Medicine Charkiv) | 1 |
| Hungary (University for Veterinary Medicine Budapest) | 9 |
| USA (University of Tennessee, Knoxville) | 1 |
| Total | 44 |

**S4 Table: Places and absolute numbers of participants graduated in a foreign country.**
