# Supplementary material for: Application of veterinary naturopathy and complementary medicine in small animal medicine—A survey among German veterinary practitioners
Source: PLoS One. 2022 Feb 28;17(2):e0264022. doi: 10.1371/journal.pone.0264022 (PMC8884514; doi:10.1371/journal.pone.0264022)
Supplement: S5 Table — (DOCX) [file pone.0264022.s005.docx]

| **Qualification** | **Incidence [n; %]** |
| --- | --- |
| Specialist veterinarian (FTA) in total | 82 (33.5 %) |
| FTA small animal science | 53 |
| FTA surgery (small animals) | 2 |
| FTA cattle | 2 |
| FTA pigs | 2 |
| FTA horses | 2 |
| FTA reproductive medicine | 2 |
| FTA internal medicine (small animals) | 3 |
| FTA laboratory medicine | 1 |
| FTA physiology | 1 |
| FTA general medicine | 1 |
| FTA poultry | 1 |
| FTA microbiology | 1 |
| FTA behavioral science | 1 |
| FTA puplic veterinary science | 1 |
| FTA nutrition | 1 |
| FTA anatomy | 1 |
| FTA chiropractic (austria) | 2 |
| FTA without naming area | 3 |
| Degree of further education total | 34 (13.9 %) |
| Ophthalmology | 2 |
| Homeopathy | 15 |
| Acupuncture | 6 |
| Behavioral science | 2 |
| Dentistry | 2 |
| Equine sports | 1 |
| Small mammals | 1 |
| Reptils | 1 |
| Manual therapies / Physiotherapy | 4 |
| IVAS – Certification | 5 (2.0 %) |
| IVCA – Certification | 3 (1.2 %) |
| ACVIM – Certification | 1 (0.4 %) |
| IAVC – Certification | 1 (0.4 %) |
| GPCert | 6 (2.4 %) |
| Master of small animal science | 5 (2.0 %) |
| Doctor´s title | 20 (8.2 %) |

**S5 Table: Frequently named qualifications / titles of participants*.**

* multiple choices possible, relative numbers calculated for population of 245 not blank answers

Legend:

ACVIM American College of Veterinary Internal Medicine

IAVC International Academy of Veterinary Chiropractic

IVAS International Veterinary Acupuncture Society

IVCA International Veterinary Chiropractic Association

GPcert General Practitioner Certificat
