# Supplementary material for: Application of veterinary naturopathy and complementary medicine in small animal medicine—A survey among German veterinary practitioners
Source: PLoS One. 2022 Feb 28;17(2):e0264022. doi: 10.1371/journal.pone.0264022 (PMC8884514; doi:10.1371/journal.pone.0264022)
Supplement: S6 Table — (DOCX) [file pone.0264022.s006.docx]

**S6 Table: Treatment modalities with potential disadvantages as seen by veterinarians.***

| **Treatment modality** | **[n]** | **[%]** |
| --- | --- | --- |
| Classic homeopathy | 333 | 78.4 |
| Complex homeopathy | 265 | 62.4 |
| Bach flower remedies | 167 | 39.3 |
| Traditional Chinese medicine | 128 | 30.1 |
| Homotoxicology | 115 | 27.1 |
| Organotherapy | 114 | 26.8 |
| Hirudotherapy (diverting therapies) | 113 | 26.6 |
| Biophysical therapies | 110 | 25.9 |
| Phytotherapy | 100 | 23.5 |
| Neural therapy | 92 | 21.6 |
| Manual therapies | 90 | 21.2 |
| Other | 13 | 3.1 |
| No answer** | 445 | - |

* multiple choices possible, relative numbers calculated for the population of 425 not-missing answers

** [n] of 870 questionnaires; [%] not calculated, as given percentages in the table refer to the population of respondents for the item
