# Supplementary material for: Application of veterinary naturopathy and complementary medicine in small animal medicine—A survey among German veterinary practitioners
Source: PLoS One. 2022 Feb 28;17(2):e0264022. doi: 10.1371/journal.pone.0264022 (PMC8884514; doi:10.1371/journal.pone.0264022)
Supplement: S7 Table — (DOCX) [file pone.0264022.s007.docx]

**S7 Table: Treatment modalities with potential advantages as seen by veterinarians.***

| **Treatment modality** | **[n]** | **[%]** |
| --- | --- | --- |
| Classic homeopathy | 205 | 45.1 |
| Complex homeopathy | 210 | 46.2 |
| Bach flower remedies | 107 | 23.5 |
| Traditional Chinese medicine | 218 | 47.9 |
| Homotoxicology | 108 | 23.7 |
| Organotherapy | 111 | 24.4 |
| Hirudotherapy (diverting therapies) | 107 | 23.5 |
| Biophysical therapies | 165 | 36.3 |
| Phytotherapy | 201 | 44.2 |
| Neural therapy | 115 | 25.3 |
| Manual therapies | 227 | 49.9 |
| Other | 12 | 2.6 |
| No answer** | 415 | - |

* multiple choices possible, relative numbers calculated for population of 455 not blank answers

** [n] of 870 questionnaires; [%] not calculated, as given percentages in the table refer to the population of respondents for the item
