# Supplementary material for: Application of veterinary naturopathy and complementary medicine in small animal medicine—A survey among German veterinary practitioners
Source: PLoS One. 2022 Feb 28;17(2):e0264022. doi: 10.1371/journal.pone.0264022 (PMC8884514; doi:10.1371/journal.pone.0264022)
Supplement: S1 File — (PDF) [file pone.0264022.s008.pdf]

JUSTUS-LIEBIG-UNIVERSITÄT GIESSEN

Fachbereich 10
Klinikum Veterinärmedizin

Justus-Liebig-Universität Gießen - Frankfurter Str. 106 - 35392 Gießen

Klinik für Geburtshilfe, Gynäkologie und Andrologie der Groß- und Kleintiere mit Tierärztlicher Ambulanz
Prof. Dr. Axel Wehrend
Dipl. ECAR
Fachtierarzt für Reproduktionsmedizin
☎ 0641-99-38700
Fax: 0641-99-38709
✉ axel.wehrend@vetmed.uni-giessen.de
Frankfurter Str. 106
D-35392 Gießen

Naturheilkundliche und komplementärmedizinische Methoden in der Veterinärmedizin - Eine Studie zur Anwendungssituation von Tierärzten/-innen

Sehr geehrte Kollegen/-innen,
dieser Fragebogen wurde im Rahmen einer Dissertation am Klinikum der Veterinärmedizin der Justus-Liebig-Universität Gießen erstellt (Betreuung durch Herrn Prof. Dr. Wehrend). Unterstützt wird die Arbeit von der Karl und Veronica Carstens-Stiftung in Essen.
Der Fragebogen richtet sich an im Klein- und Heimtierbereich tätige Tierärzte/-innen und soll wertfrei Daten zum momentanen Umgang mit naturheilkundlichen und komplementärmedizinischen Verfahren im Praxisalltag erfassen. Ich würde Sie bitten sich einige Minuten Zeit zum Ausfüllen des Fragebogens zu nehmen.
Die zugrunde liegende Zuteilung der veterinärmedizinischen Methoden in dieser Studie orientiert sich am humanmedizinischen Sprachgebrauch. So werden der Naturheilkunde u.a. Phytotherapeutika, biophysikalische Verfahren (z.B. Lasertherapien) oder auch die manuelle Therapie zugeordnet. Komplementärmedizinische, also „ergänzende" Methoden, umfassen Verfahren wie die Akupunktur, Homöopathie oder Neuraltherapie. Es ist bewusst, dass hinsichtlich dieser Zuteilungen unterschiedliche Auffassungen existieren.
Ich würde mich freuen, wenn Sie sich an dieser Studie beteiligen. Selbstverständlich werden alle Daten anonym erfasst und ausschließlich zum Zweck der Dissertation verwendet. Eine Onlinevariante des Fragebogens steht unter **http://vetmed.limequery.com/538872?lang=de** zur Verfügung. Schriftlich beantwortete Bögen können Sie an +49 7053 3276 faxen, an die Schwarzwald Tierklinik (Bühlstraße 5-7, 75387 Neubulach) oder an die E-Mailadresse istanossek@web.de weiterleiten.

Vielen Dank für Ihre Mithilfe,

mit freundlichen Grüßen

Ines Stanossek

1 Angaben zur Person

1.1 Alter: ____

1.2 Geschlecht: □ männlich □ weiblich

1.3 Landestierärztekammer in der praktiziert wird: ___________

1.4 Veterinärmedizinstudium abgeschlossen an der Universität _______________

1.5 Praktizieren Sie □ im ländlichen Raum ? □ im städtischen Raum ?

1.6 Art der Tätigkeit: □ Angestellte/r □ Praxiseigner/in □ Andere: _______

1.7 Tätigkeitsfeld: □ < 50% Klein-/Heimtiere □ ≥ 50% Klein-/Heimtiere

1.8 Verfügen Sie über veterinärmedizinische Zusatzbezeichnungen/ Fachtierarzttitel oder andere Bildungsabschlüsse?

□ nein □ ja, folgende: __________________

2 Anwendung von Verfahren der Komplementärmedizin und Naturheilkunde

2.1 Interessieren Sie sich für die Themen Komplementärmedizin und Naturheilkunde in der Veterinärmedizin?

□ nein □ ja □ keine Angabe

2.2 Warum interessieren Sie sich / Sie sich nicht für diese Thematik?

□ keine Angabe □ weil: _________________

2.3 Welche Verfahren setzen Sie in Ihrer praktischen Tätigkeit ein? In welchen Bereichen nutzen Sie diese Therapien (Mehrfachnennungen möglich)

□ ich wende keine naturheilkundlichen/ komplementärmedizinischen Verfahren an

□ ich wende folgende Verfahren in folgendem Bereich an (bitte kreuzen Sie an):

**Verfahren** / **Bereich**	Geriatrie	Onkologie	Stoffwechsel-erkrankungen	Parasitosen	Dermatologie	Infektions-erkrankungen	Reproduktions-medizin	Verhaltens-auffälligkeiten	Orthopädie	Sonstiger Bereich:
klassische Homöopathie										
homöopathische Komplexmittel										
Phytotherapeutika										
Traditionelle Chinesische Medizin (z.B. Akupunktur)										
biophysikalische Therapien (z.B. Laser, Magnetfeld, Ultraschall)										
manuelle Therapien (inkl. Chiropraxis, Massage, Osteopathie)										
ausleitende Verfahren (z.B. Blutegeltherapie)										
Bachblütentherapie										
Neuraltherapie										
Homotoxikologie										
Organotherapie										
Sonstige Verfahren: ______________________										

3 Nachfragesituation

3 Wie beurteilen Sie die Nachfrage nach oben genannten Verfahren seitens der PatientenbesitzerInnen in Ihrem Praxisalltag in den letzten 5 Jahren?

□ sinkend □ gleichbleibend □ steigend □ kann ich nicht einschätzen

4 Informationswege

4.1 Welche Wege der Informationsbeschaffung nutzen Sie für komplementärmedizinische und naturheilkundliche Inhalte?

□ Keine/ Das Thema interessiert mich nicht
□ Internet
□ Fachzeitschriften/-bücher
□ durch die ATF oder Tierärztekammern anerkannte Fort- und Weiterbildungen
□ Fortbildungen für Tierärzte/-innen anderer Art (z.B. Naturheilpraxis)
□ Fortbildungen für Nicht -Tierärzte/innen anderer Art (z.B. Naturheilpraxis humanm.)
□ Firmeninformationen (inkl. Messen)
□ Kollegen/Kolleginnen
□ Inhalte im Studium
□ Andere: _____________

4.2 Halten Sie die angebotenen Informationen (insbesondere Fort- und Weiterbildungen) im Hinblick auf die Nachfrage seitens der PatientenbesitzerInnen für ausreichend?

□ ja □ eher ja □ eher nein □ nein □ kann ich nicht beurteilen

5 Probleme und Potentiale in der Anwendung komplementärmedizinischer und naturheilkundlicher Verfahren

5.1.1 Wo sehen Sie kritische Punkte in der Anwendung komplementärmedizinischer und naturheilkundlicher Methoden im Praxisalltag? (Mehrfachnennungen möglich)

□ Keine
□ Quantitativ mangelhafte Informationen
□ Qualitativ mangelhafte Informationen
□ Unklare Studienlage/Evidenz zu Wirkung und Wirkungsmechanismen
□ Erwartungshaltung der Patientenbesitzer/-innen
□ Wechselwirkung mit anderen Verfahren/Behandlungen
□ Fehlender zeitlicher Rahmen im Praxisalltag
□ Andere: _____________

5.1.2 Bei welchen genannten Verfahren (s. 2.3) sehen Sie diese Kritikpunkte besonders?

5.2.1 Welche Potentiale sehen Sie in der Anwendung komplementärmedizinischer und naturheilkundlicher Verfahren in der Tiermedizin? (Mehrfachnennungen möglich)

□ Keine
□ Erweiterung des Behandlungsspektrums
□ Nebenwirkungsärmere Behandlungsoptionen
□ Erhöhte Kundenzufriedenheit/Kundenbindung
□ Erweiterte monetäre Potentiale (Umsatzsteigerung etc.)
□ eigene therapeutische (Arbeits-) Zufriedenheit
□ Andere: _____________

5.2.2 Bei welchen der genannten Verfahrer (s. 2.3) sehen Sie diese Potentiale explizit?

____
