# Supplementary material for: Application of veterinary naturopathy and complementary medicine in small animal medicine—A survey among German veterinary practitioners
Source: PLoS One. 2022 Feb 28;17(2):e0264022. doi: 10.1371/journal.pone.0264022 (PMC8884514; doi:10.1371/journal.pone.0264022)
Supplement: S2 File — (PDF) [file pone.0264022.s009.pdf]

1 Personal data

1.1 Age: ____

1.2 Gender: □ Male □ Female

1.3 State Veterinary Association: ____________

1.4 Place of final graduation ____________

1.5 You work: □ in rural space? □ in urban space?

1.6 Employment: □ Employee □ Self-employed □ Other: _______

1.7 Treated animals: □ < 50% small animals □ ≥ 50% small animals

1.8 Do you bear any other veterinary title or further education?

□ No □ Yes: __________________________

2 Use of naturopathy and complementary medicine

2.1 Are you interested in veterinary naturopathy and complementary medicine?

□ No □ Yes □ No statement

2.2 Why are you (not) interessted in these themes?

□ No statement □ Because of: _________________

2.3 Which treatment modalities do you practically use? In which medical fields do you use them? (Multiple choices are possible)

□ I don`t use veterinary naturopathy or complementary medicine

□ I use the following treatment modalities in the following indications (Please cross fields):

**Indication** / **Treatment**	Geriatrics	Oncology	Metabolic diseases	Parasitic diseases	Dermatology	Infectious dseases	Reproductive medicine	Behavioural disease	Orthopaedics	Other field:
Classic homeopathy										
Complex homeopathy										
Phytotherapy										
Traditional Chinese Medicine (incl. Accupuncture)										
Biophysical treatments (incl. Laser, magnetic fields, ultrasound)										
Manual treatments (incl. Chiropractic, massages, Osteopathy)										
Hirudotherapy (Diverting treatments)										
Bach flower remedies										
Neural therapy										
Homotoxicology										
Organotherapy										
Other treatment options: ______________________										

3 Demand for veterinary naturopathy and complementary medicine

3 How do you asses owner demand for veterinary naturopathy and complementary medicine over the last 5 years?

□ decreasing □ stable □ increasing □ I cannot assess

4 Information pathways

4.1 Which information pathways do you use for veterinary naturopathy and complementary medicine?

- □ none / I´m not interested
- □ Internet
- □ Scientific journals / books
- □ Acknowledged further education (ATF acknowledged)
- □ Other further education for veterinarians
- □ Other further education for non-veterinarians (e.g. human non-medical practitioners)
- □ Company information (incl. Trade fairs)
- □ Colleagues
- □ Informations within veterinary course of studies
- □ Other: ______________

4.2 Do you asses offered information as adequate regarding the demand of owners (especially further education for veterinarians)?

□ Yes □ Rather yes □ Rather no □ No □ I cannot assess

5 Disadvantages and advantages using veterinary naturopathy and complementary medicine

5.1.1 Where are challenging points for you in practically using veterinary naturopathy and complementary medicine (multiple choice possible)?

- □ None
- □ Quantitative deficient informations
- □ Qualitative deficient informations
- □ ambiguous study situation / evidence on effect and mode of action
- □ Expectations of owners
- □ Interdependence with other treatment options
- □ Lack of time in daily routines
- □ Other: ______________

5.1.2 In case of which treatment option (see 2.3) you see disadvantages the most?

____

5.2.1 Where are advantages for you in practically using veterinary naturopathy and complementary medicine (multiple choice possible)?

- □ None
- □ Expansion of treatment options
- □ Treatment modalities with less side effects
- □ Higher customer satisfaction
- □ Extended monetary potential (e.g. increase in turnover)
- □ Higher job satisfaction for yourself
- □ Other: ______________

5.2.2 In case of which treatment option (see 2.3) you see advantages the most?

____
